# Associations between rural hospital closures and acute and post‐acute care access and outcomes

**DOI:** 10.1111/1475-6773.14426

**Published:** 2024-12-30

**Authors:** Geoffrey J. Hoffman, Jinkyung Ha, Zhaohui Fan, Jun Li

**Affiliations:** ^1^ University of Michigan School of Nursing Ann Arbor Michigan USA; ^2^ Institute for Healthcare Policy and Innovation University of Michigan Ann Arbor Michigan USA; ^3^ Division of Geriatric and Palliative Medicine, Department of Internal Medicine University of Michigan Ann Arbor Michigan USA; ^4^ Center for Healthcare Outcomes and Policy, Michigan Medicine University of Michigan Ann Arbor Michigan USA; ^5^ Maxwell School of Citizenship and Public Affairs Syracuse University Syracuse New York USA; ^6^ Aging Studies Institute Syracuse University Syracuse New York USA

**Keywords:** access, acute inpatient care, health equity, long‐term care, Medicare, racial/ethnic differences in health and health care, rural health

## Abstract

**Objective:**

To determine whether rural hospital closures affected hospital and post‐acute care (PAC) use and outcomes.

**Study Setting and Design:**

Using a staggered difference‐in‐differences design, we evaluated associations between 32 rural hospital closures and changes in county‐level: (1) travel distances to and lengths of stay at hospitals; (2) functional limitations at and time from hospital discharge to start of PAC episode; (3) 30‐day readmissions and mortality and hospitalizations for a fall‐related injury; and (4) population‐level hospitalization and death rates.

**Data Sources and Analytic Sample:**

100% Medicare claims and home health and skilled nursing facility clinical data to identify approximately 3 million discharges for older fee‐for‐service Medicare beneficiaries.

**Principal Findings:**

We found that hospitals that closed compared to those remaining open served more minoritized, lower‐income populations, including more Medicaid and fewer commercial patients, and had lower profit margins. Following a closure, quarterly hospitalization rates (111.6 quarterly hospitalizations per 10,000 older adults; 95% CI: 53.4, 170.9) and average hospital lengths of stay increased (0.34 days; 95% CI: 0.13, 0.56 days). We observed no change in the average distance between patients' residential ZIP code and the hospital used (0.29 miles; 95% CI: −1.06, 1.64 miles); average number of standardized ADL limitations at PAC (0.08 SDs from the pre‐closure average; 95% CI: −0.12, 0.28 SDs); or PAC time to start (0.02 days; 95% CI: −1.2, 1.2 days). Among more isolated hospitals, closures were associated with an increase in the likelihood of readmission (0.10 percentage‐points; 95% CI: 0.00, 0.19).

**Conclusions:**

Closures were not associated with notably worsened health care access, function, or health, potentially because closures triggered care delivery adjustments involving increased numbers of patients seeking out higher quality care.

**What is known on this topic**

- Rural hospitals represent nearly half of hospitals nationwide, house one‐sixth of inpatient beds, and provide essential services to populations that are older, lower‐income, and disabled
- A hospital closure can disrupt access to acute care and shift additional strains onto the remaining rural health care infrastructure
- Hospital closures may also have implications for equity, as they occur more often in markets with high proportions of racial and ethnic minorities and Medicaid beneficiaries

**What this study dds**

- Rural hospitals that closed in the period after Medicaid expansion served more socioeconomically disadvantaged patient populations
- However, rural hospital closures were generally associated with only modest if any impediments to care access
- Closures were not associated with notably worse follow‐up health outcomes, including among minoritized beneficiaries, perhaps due to substantial increases in hospitalizations.

## 1 INTRODUCTION

Rural hospitals, 40% of which are located in the South,^1^ represent nearly half of hospitals nationwide, house one‐sixth of inpatient beds, and provide essential services to populations that are generally older, lower‐income, and with high levels of disability.^2^ From 2013 to 2017, 64 rural hospitals closed.^2^ Such closures are often the culmination of stressors such as underlying community poverty and uninsurance, shrinking patient populations, staffing shortages, and long‐standing, unstable revenue streams.^1^, ^3^, ^4^ A hospital closure can disrupt access to acute care within a community and may shift additional strains onto the remaining rural health care infrastructure, potentially increasing emergency, primary, and specialty care loads.^1^, ^5^, ^6^, ^7^ Hospital closures may also have implications for equity, as they occur more often in markets with high proportions of racial and ethnic minorities and Medicaid beneficiaries.^8^, ^9^, ^10^

Prior investigations show mixed effects of hospital closures on access and health outcomes.^11^, ^12^ Some studies have observed negative impacts of closures including longer emergency transport time^13^ and poorer adult and infant health outcomes.^6^, ^14^ For instance, closures increased the activation time for emergency responders by 7.2 min, in one study.^13^ Others have observed negative effects only for disadvantaged populations,^5^ and hypothesized that this is due to patient travel distance being driven by the perceived costs and benefits of hospital care, which in turn depend upon patient characteristics.^15^ One study found a slight increase in inpatient mortality after rural hospital closures, with larger effects for minoritized populations.^6^ However, other studies show no effect on travel distances with the exception of certain low‐income populations,^5^ and no impact on hospitalization or mortality rates.^16^ Yet other work suggests that closures may actually improve care, as additional travel times are offset by care at higher‐quality hospitals.^14^, ^17^, ^18^ Prior work has not addressed a number of methodological concerns, however. First, closures are staggered and traditional designs that fail to account for staggering may produce biased results. Second, prior work has ignored background factors that could produce compositional changes in the health of the population, the hospitalized population, and the supply of health care. Third, a limited set of health outcomes has been explored; the existing literature has primarily explored access and inpatient mortality, but has not looked at longer‐term outcomes, such as readmissions, fall injuries, and mortality occurring within 30 days of hospital discharge.

Moreover, prior studies ignore potentially important effects later along the care continuum, including the increasingly important post‐acute care (PAC), i.e., skilled nursing facility (SNF) and home health care (HHC), which are received by one‐third of hospitalized patients.^19^ Medicare generally requires beneficiaries to have a hospital stay of 3 nights or longer in order to quality for PAC referrals and reimbursement. Therefore, hospital closures may have a trickle‐down effect, in which beneficiaries either delay or do not go to the hospital after a closure. This could result in patients entering PAC with higher levels of disability due to delayed hospital care. If, in turn, PAC providers are unable to increase care provision in response, patients may have worse readmissions, fall injury, and longer‐term mortality rates after closures.

We investigated the effects of hospital closures on access and health across hospital and PAC use among Medicare beneficiaries residing in rural counties from 2014 to 2018. We examined this period given the accelerated rate of hospital closures, primarily in rural areas, following Medicaid expansion in 2014.^2^, ^4^, ^8^, ^20^ We first evaluated whether beneficiaries in rural counties with hospital closures experienced changes in number of hospitalizations and travel distance to hospitals to detect changes in access to acute care. To understand whether hospitals adjusted care delivery in response to potential care delays, we next evaluated for changes in hospital lengths of stay. Second, because a substantial portion of patients hospitalized receive PAC, we focused on patients referred to post‐acute SNF and HHC to determine whether residing in a county with a hospital closure resulted in changes in functional difficulties at PAC time to start and delays to PAC services following a hospital discharge. Third, we evaluated changes in patient health and functional outcomes among hospitalized individuals, measured with 30‐day hospital readmissions, fall‐related injuries, and 30‐day mortality. We further assessed whether closures were differentially associated with each set of outcomes for minoritized patient populations. With this approach, our findings offer policymakers novel insights into the effects of rural hospital closures on older adult access and health across the acute and PAC care continuum.

## 2 METHODS

### 2.1 Study population and data

We started by restricting to rural counties, according to the definition used by the Federal Office of Rural Health Policy and adopted by the UNC Cecil G. Sheps Center, which has kept an authoritative list of rural closures since January 2005.^21^ This rural definition includes areas with Rural Urban Community Area (RUCA) codes of 4 or greater, identifying isolated, small, and large (less populated areas in urban counties) rural areas.^22^ It additionally includes several non‐rural (metro) census tracts and counties if they are sparsely populated or without an urbanized area. Using the Sheps list, we obtained hospital locations and closure dates from each closed hospital. Each rural county was then designated as having a closure based on the first incidence of closure during 2015–2017. This resulted in 1556 unique counties, of which 1545 (99%) were rural. We identified 32 hospital closures, 10 of which were in non‐rural counties. Closures entailed complete (no longer providing any healthcare services) and converted closures (no longer providing inpatient care but continuing to provide some health care services, such as primary care or skilled nursing care). They also involved short‐term acute hospitals and Critical Access Hospitals (CAH).

Our discharge‐level sample consisted of discharges among older (65 years and older) FFS Medicare beneficiaries residing in short‐term acute hospitals located in rural counties in the 50 U.S. states. We used 100% Medicare claims data from the MedPAR file to identify hospitalizations from 2015 to 2017. Hospitalization claims that data were then linked to 100% samples of each of the Outcome and Assessment Information Set (OASIS) and the Minimum Data Set (MDS), which capture standardized assessments of patient function upon the start of home health and skilled nursing facility care. To capture pre‐ and post‐closure beneficiary health care utilization, we also incorporated 100% MedPAR data (2014–2018). This resulted in 15,500,418 discharge‐level observations. We excluded non‐rural counties in the control group (counties without a closure) and discharges in a given county in the 3 months prior to a hospital closure in that county, to account for closures that take time to effectuate (i.e., a slowdown in admissions that precedes the final date of closure). This resulted in a final dataset with 3,043,101 observations and 32 rural hospital closures for our main analytic sample. The number of cohorts in each model differed. In the mortality model, there were 9 cohorts, with approximately 3.4% of observations contributing to the “treated” cohort (see Appendix S1, section 2).

We obtained county‐level characteristics from the Social Determinants of Health (SDOH) file from the Agency for Healthcare Research and Quality (AHRQ). Hospital‐level characteristics were obtained from the Center of Medicare and Medicaid Services (CMS) Impact Files and from the National Academy for State Health Policy (NASHP) hospital database.

### 2.2 Outcomes

All outcomes and covariates were assessed at the county level.

#### 2.2.1 Hospital‐related care

We examined county‐level (1) rate of hospitalizations per 10,000 older adults (65 years and older), (2) average trimmed distance to the discharging hospital, measured as the distance between a patient's residential ZIP code and the ZIP code of the discharging hospital, and (3) average length of stay during hospitalization. The trimmed distance capped distances at the 99th percentile to account for large, outlier values that could reflect patients from out‐of‐state and not local commuting patterns. To assess whether rural hospital closures were associated with changes in extreme outcomes, we separately evaluated the share of each county's beneficiaries experiencing a particularly long distance to the hospital or hospital length of stay (i.e., equal to or exceeding the top tertile of distance or length of stay of the pre‐closure period).

#### 2.2.2 PAC‐related care

For the subsample of older FFS Medicare beneficiaries with PAC use beginning within 2 weeks of a hospital discharge, we examined county‐level: (1) average number of activities of daily living (ADL) at PAC time to start (see Appendix S1) and (2) average time between hospital discharge and start of PAC, measured as the number of days between hospital discharge and the admission date of the SNF or first home health visit. Our main PAC analyses involved only hospitals without swing beds. Given that the time between hospital discharge and start of PAC for those hospitals should be minimal, we would not expect increases in either of the two PAC outcomes.

We also assessed county prevalence of extremes, or the shares of PAC discharges with particularly large values (i.e., equal to or exceeding the top tertile of values in the pre‐closure period).

#### 2.2.3 Health outcomes for hospitalized individuals

We examined any 30‐day: (1) all‐cause hospital readmissions, (2) mortality, and (3) hospitalization for a fall‐related injury (FRI). FRIs were identified using a previously validated algorithm.^23^ These three outcomes are considered measures of quality of care for older adults during and after hospital discharge.^20^, ^24^, ^25^

#### 2.2.4 Population outcomes regardless of hospitalization

We also explored whether there were changes in the underlying population, and not merely among hospital users. To do so, we examined each of county‐level population size, rates per 10,000 of death, primary care physicians (PCP), and numbers of rural health clinics, and preventable hospitalizations per 10,000 Medicare beneficiaries.

### 2.3 Sub‐populations

We re‐estimated models for individuals identified in the MedPAR dataset as minoritized (i.e., Black, Hispanic, or Other), which we hereafter refer to as “minoritized” patients or populations. Starting with the discharge‐level data, we excluded White patients, and then again collapsed the data to create a county‐quarter level dataset for our analyses.

### 2.4 Analysis

We first described the rural hospitals that closed compared to those that did not close. We focused on population characteristics (e.g., county population, percentage Black, Hispanic, and Asian, uninsured); hospital characteristics (e.g., bed size, census, safety‐net, and teaching status); financial characteristics (profit margins, revenue and expenses, payer mix); and hospital patient characteristics (e.g., comorbidities, percentage male, 85+, Black, Hispanic, dual eligible, urgent cases).

We next assessed the impacts of closures. In this study, we used the augmented inverse probability weighting (AIPW) estimator^26^ to model the outcome and treatment in a difference‐in‐differences design. This method is also called doubly robust because of the fact that as long as either the outcome or treatment models are correct, then the estimates of treatment effects would be consistent.^27^ This is an advantage compared to other estimators, such as two‐way fixed effects (requires correct outcome model),^28^ the outcome regression approach (requires correct outcome model), and inverse probability weighted estimators (requires correct treatment model).^29^, ^30^

The AIPW difference‐in‐differences model is estimated flexibly as opposed to using a single model. The model structures of the AIPW can be broadly defined as (1) specifying a propensity score model for the treatment to obtain probability weights, (2) applying linear regression to estimate average outcomes, and then (3) combining components to obtain estimates of treatment effects. Steps 1–2 are conducted for each cohort and time. In (1), the propensity score uses a logit to model the probability of an observation being in the treatment group at a given period as a function of pre‐treatment covariates: a county's population, median household income, rate of primary care physicians, percentages of population that is Black, that is enrolled in Medicare, that is uninsured; the profit margin of the hospital with the lowest profit margin in a county in a given year; whether a hospital had the lowest annual census (number of discharges) of all hospitals in a county in a given year; the Medicaid payor mix of the hospital with the highest Medicaid mix in a given year; and an indicator for a hospital in a county having two or more of the lowest profit margin, lowest annual census, and highest Medicaid mix of all hospitals in a county in a given year. We use this model to obtain probability weights. In (2), the linear regression model for outcomes uses OLS to model the average outcome for treatment and comparison groups at a given period controlling for pre‐treatment covariates involving beneficiary demographics and health (age, sex, race/ethnicity, and number of comorbidities). Then, in (3), we combine average outcomes and probability weights using the potential outcomes framework. Standard errors are computed recognizing within‐county correlation using the approach proposed in Callaway and Sant'Anna (2021) (influence‐function).^26^ Standard errors were clustered at the county level to control for correlation between measurements within counties.

The difference‐in‐differences estimator has several key assumptions.^26^ The parallel trends assumption requires that the average conditional outcomes of the treated and untreated groups would be similar in the absence of treatment. The overlap assumption requires that at least a small fraction of the population should be treated and there should be some probability of non‐treatment for every value of the covariates. Additionally, the model assumes that once the units receive treatment, they remain treated (i.e., now new hospitals opened in counties with closures), which is the case with our data, and that closures are exogenous (i.e., that they were not a function of patient health outcomes); existing literature and our data suggest that closures are a function of local economic circumstances and hospital profit margins, not patient health.^1^, ^2^, ^3^, ^8^ While parallel trends are inherently untestable, visual inspection of figures provided in the Appendix S1 suggests that there is no violation, especially in periods close to the closure time, although some isolated quarters are statistically significant. These minority quarters of statistically significant violations are consistent with the Wald tests results (see Appendix S1) suggesting that not all pre‐treatment quarters had zero effects.

We re‐estimated the models to examine subgroup effects of closures for minoritized populations. We examine this population, given the structural determinants that could be driving health care supply and its use for disadvantaged patient populations.^31^

#### 2.4.1 Additional tests

To address potential changes in patient composition (i.e., sicker populations over time, after closures), we examined any changes in demographic and comorbidities after closures. For instance, if patients residing in counties with closures were older or sicker at baseline, then they may also have greater care needs at hospitalization for reasons unrelated to changes in hospital access. These changes were examined for the overall and PAC cohorts. We also examined untrimmed distance to the hospital.

We conducted several sensitivity analyses. First, we conducted separate analyses on hospitals that closed within versus at or beyond 50 miles of the nearest hospital to account for potential differences in effect according to the local supply of alternative hospital beds. Second, we examined only closures among hospitals with large Medicare populations, given that effects of closures would likely be smaller for hospitals with more limited Medicare patient volume. Third, we examined PAC outcomes only for hospitals with swing‐beds.

## 3 RESULTS

### 3.1 Characteristics of closed rural hospitals

Between 2015 and 2017, 32 hospitals closed. On average, closed hospitals had 521.2 (SD = 442.6) admissions annually 4 years before closure, which decreased to 242.2 (SD = 266.3) admissions 1 year prior to closure (Table A1). Among 11 closed hospitals that had post‐acute swing‐beds, the mean number of swing‐bed days was 78.4 (SD = 70.4) 1 year prior to closure. For the average hospital that closed, the mean distance to the nearest hospital was 17.1 (SD = 14.2) miles. The mean travel distance for hospital care for a beneficiary residing in a county with a closure in the year prior to a hospital closure in that county was 3.7 miles (SD = 2.3). That travel distance increased to 5.0 (SD = 2.6) miles in the year after a closure.

### 3.2 Comparison of admissions to nearest hospitals and other hospitals in same county after a closure

As shown in Table 1, after the 32 hospital closures, the nearest hospitals to 12 of those closed hospitals had decreases in hospital admissions in the year following those hospitals' closures, while 20 of the nearby hospitals had increased admissions. Overall, there was a decrease of 65 admissions (0.5%) at the nearest hospitals to closed hospitals in the year following closures. However, by the second year after closure, there was an increase in admissions in nearby hospitals of 877 admission (7.1%). Similarly, in the first and second years after closures, admissions increased by 15% and 32%, respectively, in the counties in which hospitals closed (Table A2).

**TABLE 1:** Descriptive statistics of rural hospitals by closure status, 2014–2018.

	Overall (Mean (SD)/%)	Closed (Mean (SD)/%)	Not closed (Mean (SD)/%)
County characteristics			
Total population	36,954.5 (33,315.5)	67,906.4 (67,787.8)	35,525.7 (32,417.8)
Percentage Black	7.2	14.8	7.0
Percentage Hispanic	8.1	11.9	8.1
Percentage Asian	0.9	0.7	0.9
Percentage aged 65 and older	18.4	17.1	18.4
PCP rate per 100,000	58.7 (29.7)	56.5 (33.5)	58.8 (29.6)
Percentage uninsured	11.8	15.3	11.8
Median household income ($)	47,331.0 (9655.2)	43,123.7 (8586.2)	47,389.3 (9658.7)
Hospital characteristics			
No. of beds	82.1 (63.2)	52.6 (29.1)	82.7 (63.6)
Average daily census	31.2 (36.9)	12.6 (11.0)	31.6 (37.2)
No. of annual discharges	1227.5 (1283.8)	496.8 (410.6)	1244.2 (1292.1)
Safety‐net provider	34.0	36.8	33.9
Teaching hospital	9.3	5.3	9.4
Readmission penalty percentage	0.56 (0.60)	0.46 (0.53)	0.57 (0.60)
VBP payment adjustment percentage	0.22 (0.43)	−0.05 (0.39)	0.23 (0.43)
Financial characteristics			
Profit margin			
Total net (%)	3.7	−11.6	3.7
Medicare (%)	−1.1	−3.0	−1.1
Medicaid (%)	−31.8	−38.8	−31.8
Commercial (%)	33.7	10.6	34.0
Net patient revenue (millions, $)	116.8 (144.9)	23.3 (14.7)	117.0 (145.0)
Operating expenses (millions, $)	118.0 (148.0)	25.5 (14.9)	117.7 (148.0)
Hospital operating costs (millions, $)	91.6 (108.0)	20.2 (10.9)	91.8 (108.1)
Net charity care cost (millions, $)	0.9 (2.8)	1.1 (1.2)	0.9 (2.8)
DSH percentage	0.29 (0.13)	0.32 (0.12)	0.29 (0.13)
Medicare payer mix (%)	35.9	33.9	35.9
Medicaid payer mix (%)	15.6	17.8	15.6
Commercial payer mix (%)	38.9	35.8	38.9
Hospital patient characteristics			
Mean comorbidity count (SD)	3.2 (1.9)	3.1 (1.8)	3.2 (1.9)
Percentage male	39.8	36.7	40.0
Percentage 85+	27.1	27.9	27.1
Percentage White	91.2	83.4	91.2
Percentage Black	5.5	14.6	5.5
Percentage Hispanic	0.6	0.8	0.6
Percentage urgent cases	10.4	7.3	10.4
Readmission rate	18.1	17.0	18.1
Mortality rate	0.3	0.3	0.3

Abbreviations: DSH, Disproportionate Share Hospital; SD, standard deviation; VBP, value‐based purchasing.

### 3.3 Comparison of rural hospitals by closure status

As shown in Table A3 in the Appendix S1, closed hospitals were in counties with larger populations (67,906 vs. 36,526), greater percentages of Black (14.8% vs. 7.0%) and Hispanic populations (11.9% vs. 8.1%), more uninsured (15.3% vs. 11.8%%) and lower household incomes ($43,134 vs. $47,389). Closed hospitals had fewer beds, lower average daily census, and more of them were safety‐net providers (38.9% vs. 22.0%). Closed hospitals had negative net profit margins (−16.2%) compared to positive ones (2.2%) for non‐closed hospitals. Profit margins were −3.0% for Medicare and −38.8% for Medicaid (compared to −1.1% and −31.8% for non‐closed hospitals).

### 3.4 Regression results

Below, we present regression results for hospital‐related outcomes, PAC outcomes, health‐related patient outcomes, population outcomes, and additional test results.

#### 3.4.1 Hospital‐related outcomes

**FIGURE 1:**
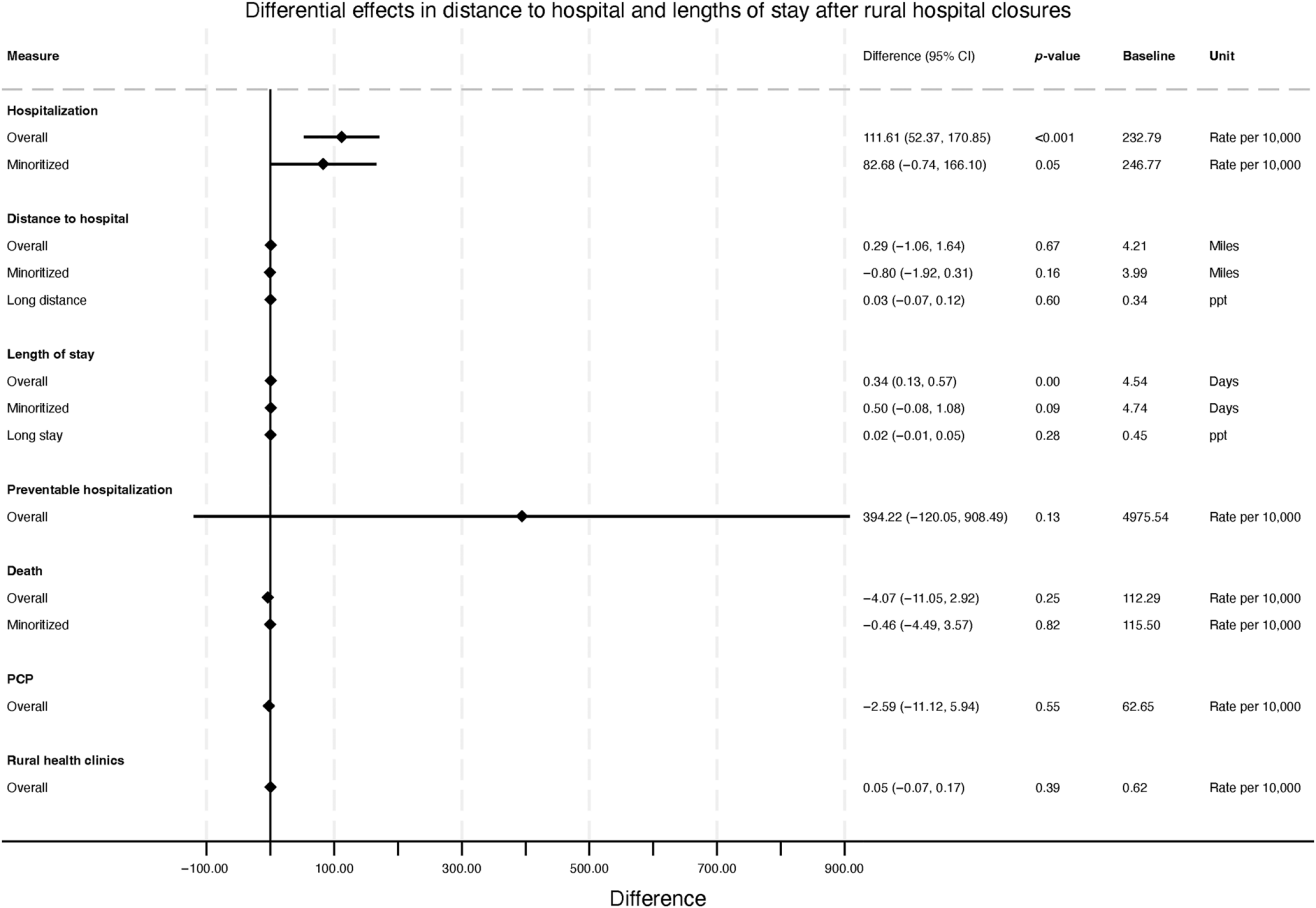
Differential changes after rural hospital closures in average and extreme patient‐level outcomes (distance to hospital, length of stay), overall and by minoritized patient status, and in population‐level outcomes. Differential changes represent changes in distance to hospital overall and for minoritized patient population admissions for areas with and without hospital closures. For “Long stay,” the difference reflects the change in the probability of a long drive to the hospital after a rural hospital closure.

We saw increases in the rate of hospitalizations and average lengths of stay, but no change in the average distance to a hospital, following rural hospital closures. Specifically, following a closure, quarterly hospitalization rates increased (an additional 111.61 quarterly hospitalizations per 10,000 older adults; 95% CI: 52.37, 170.85), from a baseline of 232.79 (SD = 114.81; Figure 1). Average hospital lengths of stay increased by 0.34 days (95% CI: 0.13 days, 0.57 days), from a baseline of 4.54 days; (SD: 4.83) for the overall population (Figure 1).

However, we observed a non‐significant increase of 0.29 miles (95% CI: −1.06 miles, 1.64 miles), from a baseline of 4.21 miles (SD = 6.18), in the average distance between patients' residential ZIP code and the hospital used (Figure 1). Closures were also not associated with an increase in the likelihood that a person had a particularly long distance of 4.90 miles or longer to get to a hospital. Specifically, the probability of driving a long distance non‐significantly increased by 0.03 percentage points [ppt] (95% CI: −0.07 ppt, 0.12 ppt; Figure 1).

#### 3.4.2 Population outcomes regardless of hospitalization

We examined population outcomes after hospital closures to see whether there were compositional shifts in population‐level health or the supply of health care that could influence the main outcomes we examined above. However, following a closure, there were no observed changes in preventable hospitalizations, population‐level death rates, PCP rates, or rural health clinic supply (Figure 1).

#### 3.4.3 PAC‐related outcomes

**FIGURE 2:**
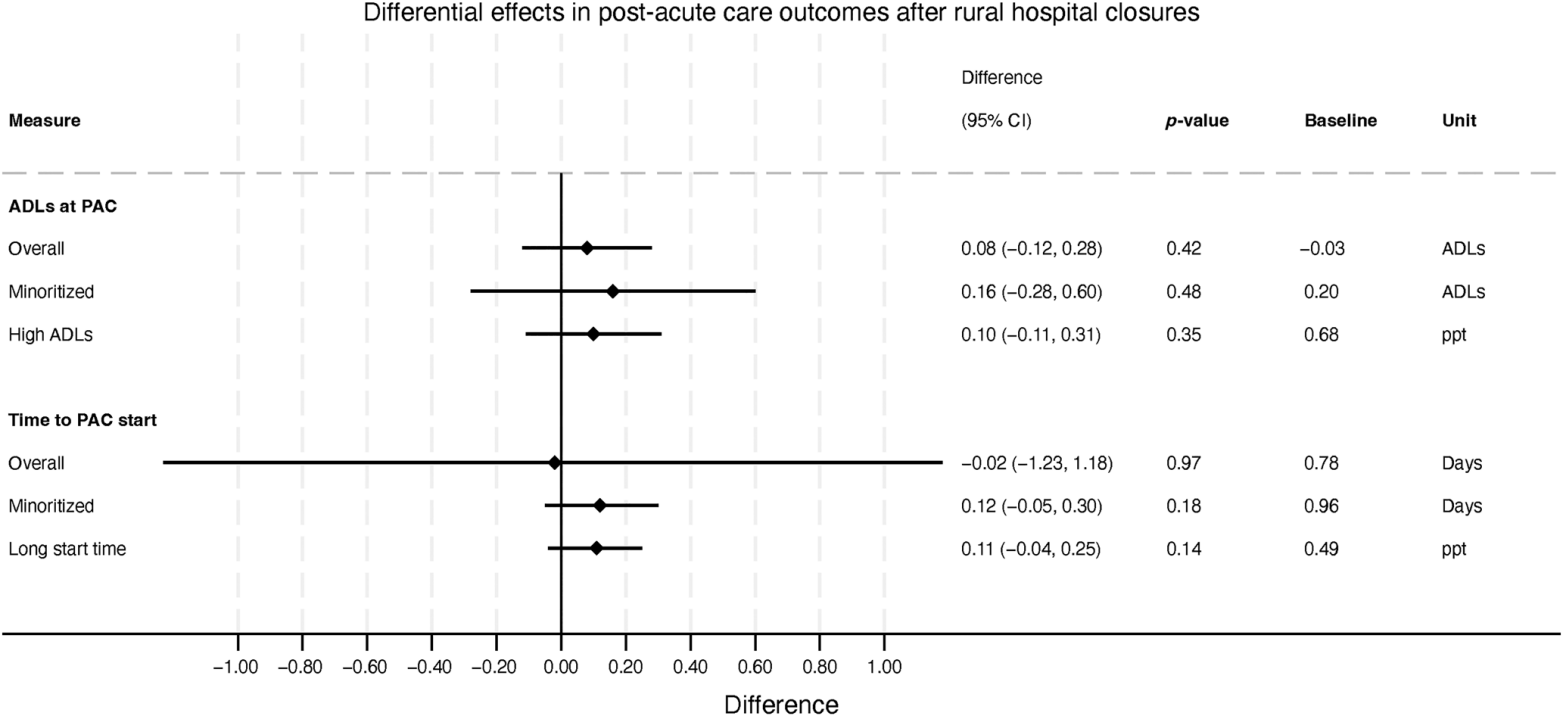
Differential changes after rural hospital closures in average and extreme patient‐level post‐acute care outcomes, overall and by minoritized patient status. Differential changes represent changes in the number of activities of daily living (ADL) limitations and days from hospital charge to the time of entry into post‐acute care (PAC) overall and for minoritized patient population admissions. For “High ADLs,” the difference reflects the change in the probability of 13 or more ADLs at PAC entry after a rural hospital closure. For “Long start time,” the difference reflects the change in the probability of long gap between hospital discharge and PAC entry after a rural hospital closure.

We did not observe changes in PAC‐related outcomes. First, after a closure, the average number of standardized ADL limitations (SDs) at PAC entry was unchanged, with a non‐significant increase of 0.08 SDs (95% CI: −0.12 SDs, 0.28 SDs) from the pre‐closure average (Figure 2). Moreover, no differences were observed after closures in the prevalence of people with a particularly high number of ADL difficulties of 13 or more at PAC time to start (0.10 SDs; 95% CI: −0.11 SDs, 0.31 SDs).

The PAC time to start (days between hospital discharge and PAC initiation) non‐significantly decreased by 0.02 days (95% CI: −1.23 days, 1.18 days), and so was not associated with closures. We also observed a non‐significant increase of 0.11 days (95% CI: −0.04 days, 0.25 days) in the prevalence of people who had long PAC waiting times.

#### 3.4.4 Health outcomes for hospitalized individuals

**FIGURE 3:**
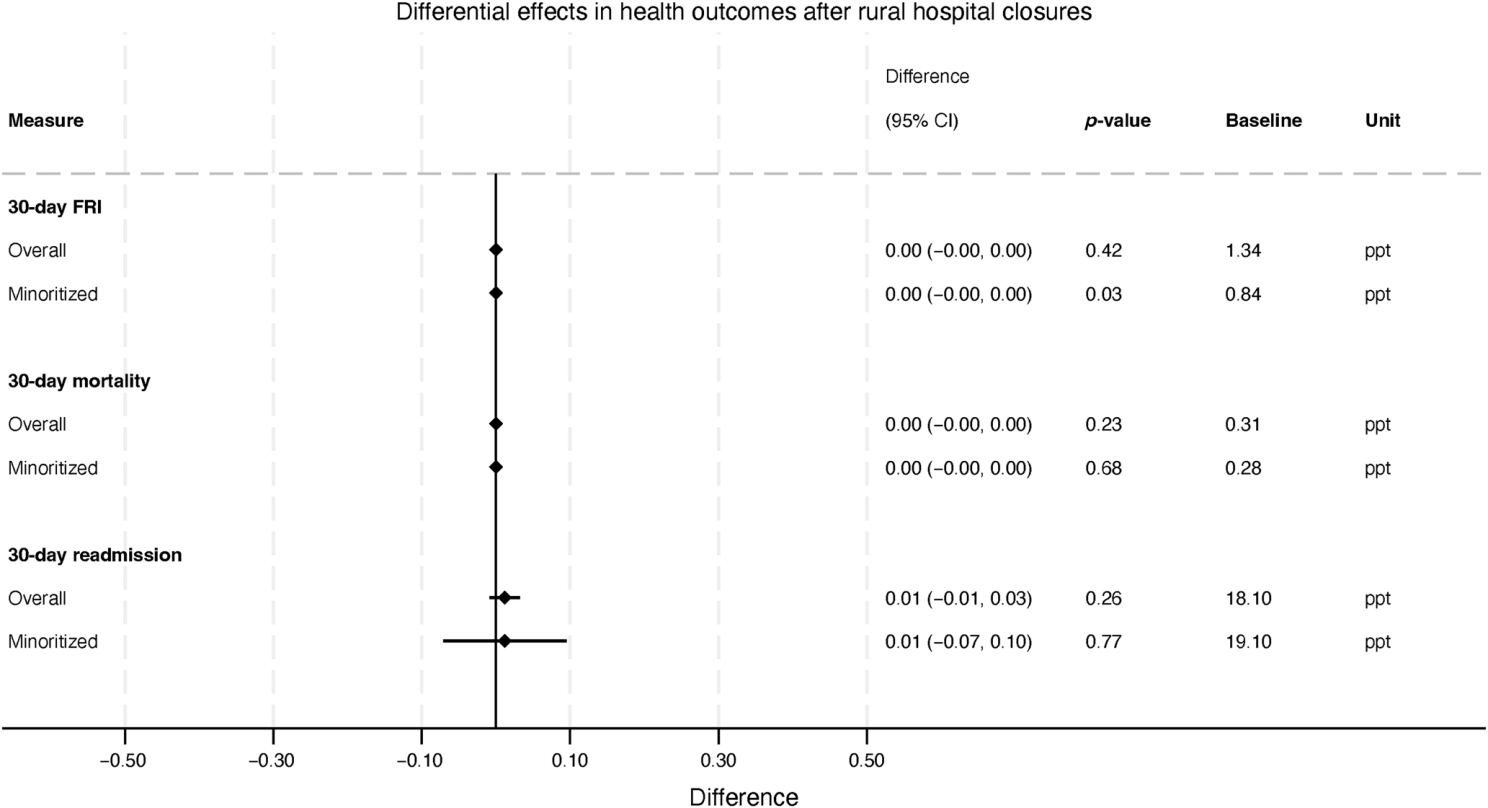
Differential changes after rural hospital closures in patient‐level health outcomes, overall and by minoritized patient status. Differential changes represent changes in the likelihood of each outcome (30‐day fall‐related injury [FRI], 30‐day mortality, and 30‐day readmission).

After hospital closures, there was no evidence of worsened (or improved) health outcomes. First, no associations between rural hospital closures and 30‐day readmissions were observed (a non‐significant increase of 0.01 percentage points [ppt]; 95% CI: −0.01 ppt, 0.03 ppt; Figure 3). Second, no change was observed in 30‐day mortality (0.00 ppt; 95% CI: −0.00 ppt, 0.00 ppt). There were also no changes in FRIs (0.00 ppt; 95% CI: 0.00, 0.00).

#### 3.4.5 Results for minoritized patient populations

For the subgroup of minoritized patient populations, there were generally no observable effects from hospital closures on the outcomes examined.

#### 3.4.6 Additional test results

To see whether there were composition shifts in the hospital and PAC patient populations after rural hospital closures, we assessed changes in patient comorbidities and other characteristics at hospitalization. We saw no changes indicative of a change in the composition of older adults in communities with closures (Figure A1). Additionally, results for untrimmed distances to the hospital were similar to those for trimmed distances (Figure A2).

We also examined several sub‐groups to assess whether any effects were restricted to hospital sub‐groups hypothetically more (or less) likely to affect patient outcomes. For the subgroup of hospitals that closed and was not within 50 miles of nearest hospital, we observed an increase of 0.10 ppt in the likelihood of a 30‐day readmission (95% CI: 0.00, 0.19; Table A4). For the subgroup of hospitals with swing beds, the average number of standardized ADL limitations at PAC entry decreased by 0.45 SDs (95% CI: −0.64 SDs, −0.25 SDs).

## 4 DISCUSSION

In this study, rural hospitals that closed in the period after Medicaid expansion served more socioeconomically disadvantaged patient populations. However, closures were generally associated with only modest, if any, impediments to care access. There was limited evidence of older Medicare beneficiaries being exposed to longer distances to get to hospitals, or that they had worsened function upon discharge. Moreover, beneficiaries did not have notably worse follow‐up health outcomes, perhaps due to substantial increases in hospitalizations. Collectively, these findings suggest that any health or equity‐related harms from rural hospital closures are mitigated by increased inpatient use at alternative hospitals.

Unlike prior work, we observed limited evidence of poorer hospital access after closure.^5^, ^11^, ^13^ Overall, patients did not see longer average distances or greater likelihood of very long distances to hospitals\after hospital closures. These findings were robust to our use of trimmed, as well as untrimmed distances, therefore accounting for outlying distances for out‐of‐state patients. It appears that patients were able to make use of nearby hospitals. By year 2 after the average hospital closure, there was a 7% increase in admissions to the nearest hospital to a closure hospital, and a 33% increase in admissions to other hospitals within the same county. In all, while patients at more isolated hospitals (>50 miles from the nearest hospital) may have experienced slightly impaired access, overall access was not severely disrupted.

We had expected that rural closures would translate to greater functional and health needs that would require care delivery adjustment by providers. However, we saw no changes in function nor in access to post‐acute services that often address functional needs. The sole exception was minoritized patients, who had longer times to the start of post‐acute care but did not have poorer function at the start of post‐acute care.

More broadly, our findings suggest that hospital closures did not worsen health for residents in counties with closures. We observed no changes in hospital‐related readmissions, mortality, or fall injuries. There were also no shifts in population‐level measures including deaths or preventable hospitalizations. Because the demographics and comorbidity levels of patients as well as population‐level measures were unchanged, and there were no changes in provider supply (rural health clinics, primary care physicians) after closures, it is unlikely that these null findings are the result of selection effects (i.e., changes in the underlying hospitalized population or provider characteristics after closures). It should be noted, however, that this failure to find evidence of an effect is not dispositive evidence that closures do no harm.

Prior work assessing health effects has often focused on mortality, with mixed findings. Joynt et al. (2015) did not see changes in population‐level or hospitalization‐level mortality, or hospitalization rates after closures, while several studies have observed mortality effects when examining hospital closures.^6^, ^18^, ^20^ We contribute to the literature using a more robust study design that accounts for the staggered timing of populations' exposures to closures. With this approach, we did not see any evidence for increased mortality or worsening of function, although we did see substantial increases in population‐level hospitalization rates after closures.

This study had several limitations. First, while our modeling approach addresses residual confounding from time‐invariant unobserved factors, we cannot rule out that time‐variant factors aside from hospital closures resulted in changes in hospital and PAC use. Any compositional shifts in patient risk or health care supply could dampen the internal validity of our study. However, we did not observe changes in total population size, physician or health clinic supply, or patient demographics and comorbidity, which could create background variation in observed rates of hospitalizations or patient risk, and therefore health outcomes. Moreover, our extensive controls for time‐variant factors and consideration for heterogeneous treatment effects improves upon earlier literature that does not account for such methodological issues like staggered timing of closures.^20^ Second, we did not assess CAHs or other smaller, rural hospitals separately from other rural hospitals given power limitations from counties with few CAH closures. Due to generous reimbursement, CAHs are less likely to close than other rural hospitals. Therefore, impacts may differ according to the type of hospital closure. Third, while the visual inspection of pre‐treatment trends suggests no violation of the parallel trends assumption, the Wald test results are consistent with a minority of pre‐treatment quarters (in our lengthy pre‐treatment period) having average treatments that are statistically significantly different than zero. Therefore, our results should be interpreted as associational and not causal.

These limitations notwithstanding, our findings have implications for policymakers. First, our work suggests that rural areas are able to accommodate hospital closures, without evidence of negative impacts on function (fall injuries, ADLs) or health (hospitalizations, mortality). It may be that patients using a smaller number of rural facilities can benefit from improved quality due to higher volumes at remaining hospitals, which mitigates the impacts of closures.^32^ Alternatively, after closures, beneficiaries may shift to care at alternative hospitals that offer equal or better quality of care,^14^, ^17^, ^18^ which reduces the harm of a closure and may even improve outcomes if lower‐quality hospitals closed.

For these reasons, policymakers may wish to reconsider rural payment structures to ensure the feasibility of providing care in areas that have high labor costs and among providers that often operate at low margins, while also ensuring value for patients and to CMS.^30^ While our findings are reassuring that existing health care infrastructure can weather closures, it is necessary to ensure that remaining providers are supported and can continue to serve these communities. This is particularly true for racially and ethnically diverse patient populations treated by more poorly financially resourced hospitals.

Second, while our work does not show strong evidence of function and health impacts from worsened access, policymakers may wish to take note of other, potential costs that may result from closures. For instance, patients may absorb new costs associated with transport (by having to identify alternative hospital care options) or those related to caregiving needs. To the extent patients are inconvenienced or health is affected in the longer term is unclear from our analyses. Given rural community hospitals' role as “safety‐net” providers, including entry points for primary care, closures can have longer‐lasting impacts beyond those measured here.^1^ Moreover, closures might increase prices, due to increased market power of remaining hospitals,^33^ or if overall hospitalizations increase, as we observed. Closures can also result in provider exodus and industry job losses that impact local economics and remaining primary and specialty care options.^34^ Finally, given closures at safety‐net hospitals serving lower‐income populations, closures may have equity implications notwithstanding limited evidence of worsened health outcomes.

## 5 CONCLUSION

We find evidence that rural communities may weather disruptions associated with hospital closures. Closures were associated with moderate worsening of hospital access but not worsened function or health for older populations, potentially because closures triggered care delivery adjustments involving increased numbers of patients seeking out higher‐quality care.

## FUNDING INFORMATION

No funding to report.

## CONFLICT OF INTEREST STATEMENT

None to report.

## Supporting information


Appendix S1.

## Data Availability

The data that support the findings of this study are available from CMS. Restrictions apply to the availability of these data, which were used under a Data Use Agreement for this study.
