# Supplementary material for: Associations between rural hospital closures and acute and post‐acute care access and outcomes
Source: Health Serv Res. 2024 Dec 30;60(3):e14426. doi: 10.1111/1475-6773.14426 (PMC12120529; doi:10.1111/1475-6773.14426)
Supplement: Supplementary file 1 — Appendix S1. [file HESR-60-0-s001.docx]

**Appendix**

1. Measurement of activities of daily living (ADL)
2. Information about staggered difference-in-differences model specifications
3. Table A1. Characteristics of closed hospitals
4. Table A2. Numbers of admissions to nearest hospital after rural hospital closure
5. Table A3. Numbers of admissions to other hospitals in same county after rural hospital closure
6. Figure A1. Differential changes in comorbidities after rural hospital closures
7. Figure A2. Differential changes in untrimmed distances to hospitals after rural hospital closures
8. Table A4. Sensitivity results for associations with rural hospital closures

1. **Measurement of activities of daily living (ADL)**

For the subsample of older FFS Medicare beneficiaries with PAC use beginning within two weeks of a hospital discharge, we examined county-level: (1) average number of activities of daily living (ADL) at PAC time to start (see Appendix) and (2) average time between hospital discharge and start of PAC, measured as the number of days between hospital discharge and the admission date of the SNF or first home health visit.

For (1), we used two separate total function scales, one each from OASIS (for home health) and MDS (for SNF). Using a previously developed approach, our OASIS scale ranged from 0-16 limitations, assessing a large set of functional limitations (e.g., with grooming, upper and lower body dressing, bathing, and feeding) ^1^. With a range of 0-24 limitations, the MDS scale assesses a larger set of largely similar limitations. In each scale, total scores were computed by translating agency-reported, activity-specific scores to scores of 0 for independence, 1 for minor assistance, and 2 for dependency, then summing across limitations. Scores were then standardized to facilitate interpretation.

2. **Information about staggered difference-in-differences model specifications and cohorts**

For the staggered difference-in-differences design, We used augmented inverse probability weights (AIPW) as our estimator for each of outcome and treatment models. Our outcome model regressed a specific outcome on average characteristics of patients (county-level averages of patient age and comorbidity scores and proportions of patients who were male, Black, and Hispanic). Our treatment model regressed a hospital’s closure on a number of county-level characteristics (median household income, PCP rate, percentages of population that are Black, enrolled in Medicare, and are uninsured, and total population). We implemented the staggered difference-in-differences design with regression adjustment using Stata’s xthdidregress command.^2^

Our time variable was an indicator for the quarter. We included 24 quarters (e.g., quarter 1 from January to March of 2014 and quarter 24 from October to December of 2019). Our group variable was an indicator for the county. Our key variable of interest was the interaction between quarter and county, where it is 1 if a given county had a closed hospital in that quarter (i.e., in any quarter after the closure), and 0 otherwise.

The number of cohorts (including treated and untreated cohorts) in each model differed. In the mortality model, there were 9 cohorts, with approximately 3.4% of observations contributing to the “treated” cohort. In the distance to hospital, there were also 9 cohorts. The treated cohorts contributed 100,829 observations to the total (or, 3.4%), while the untreated cohort contributed 2,849,002 observations.

3. **Visual illustration of parallel trends assumption**


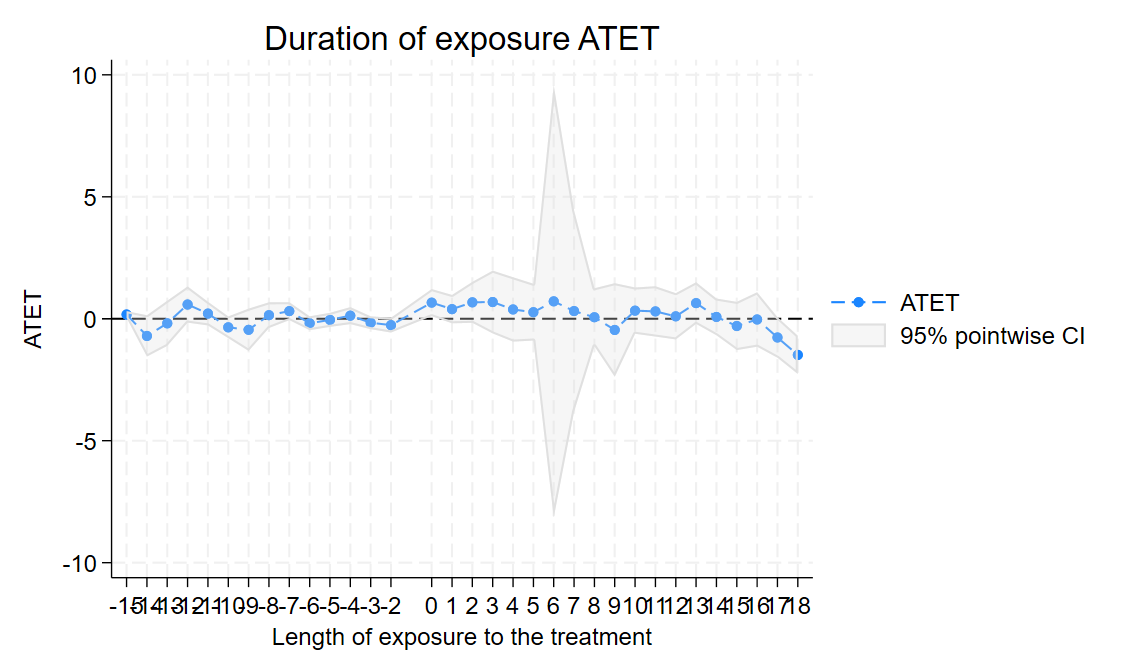
Figure 1. Dynamic graph for distance to hospital

Parallel-trends test (pretreatment time period)

H0: Treatment effects in all the pretreatment periods are zero

chi2 (61) = 13839.60

Prob > chi2 = 0.0000

Figure 2. Dynamic graph for length of stay


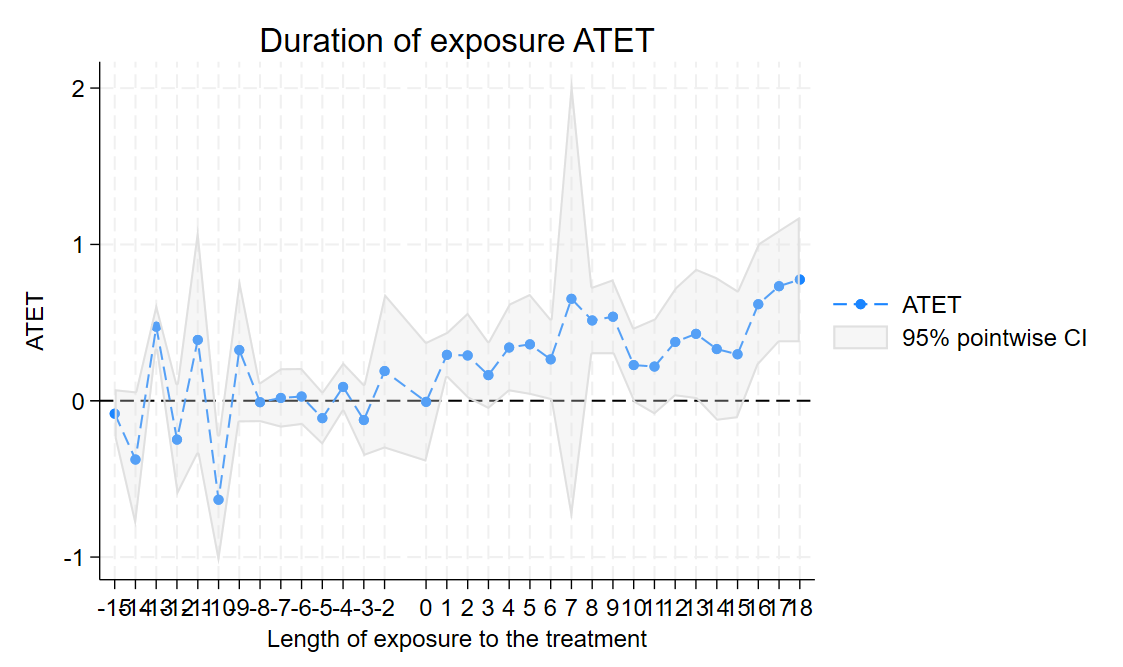


Parallel-trends test (pretreatment time period)

H0: Treatment effects in all the pretreatment periods are zero

chi2(61) = 20332.59

Prob > chi2 = 0.0000

Figure 3. Dynamic graph for distance to hospital (tertile analysis)


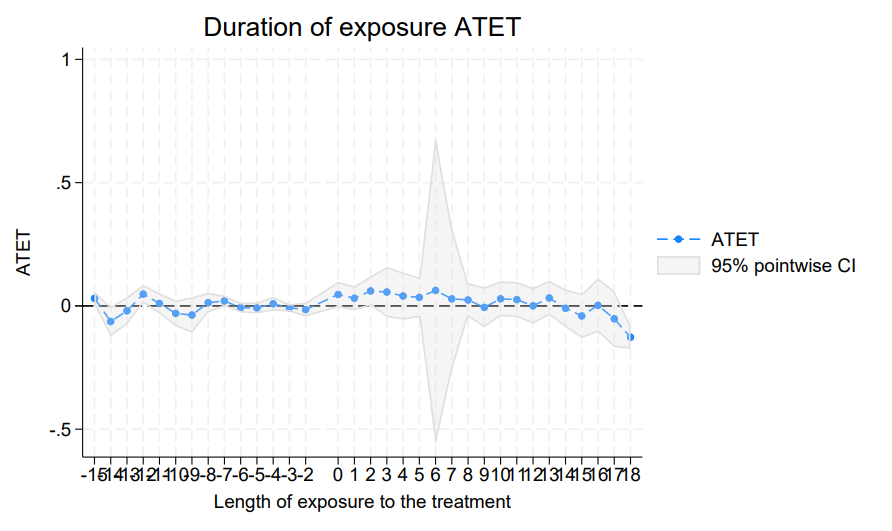


Parallel-trends test (pretreatment time period)

H0: Treatment effects in all the pretreatment periods are zero

chi2(61) = 14794.15

Prob > chi2 = 0.0000

Figure 4. Dynamic graph for length of stay (tertile analysis)
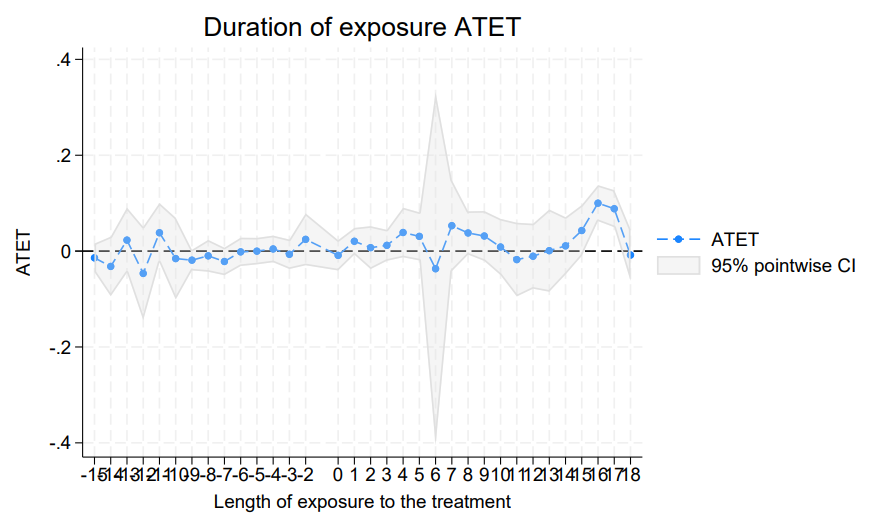


Parallel-trends test (pretreatment time period)

H0: Treatment effects in all the pretreatment periods are zero

chi2(61) = 21817.36

Prob > chi2 = 0.0000

Figure 5. Dynamic graph for 30-day readmission


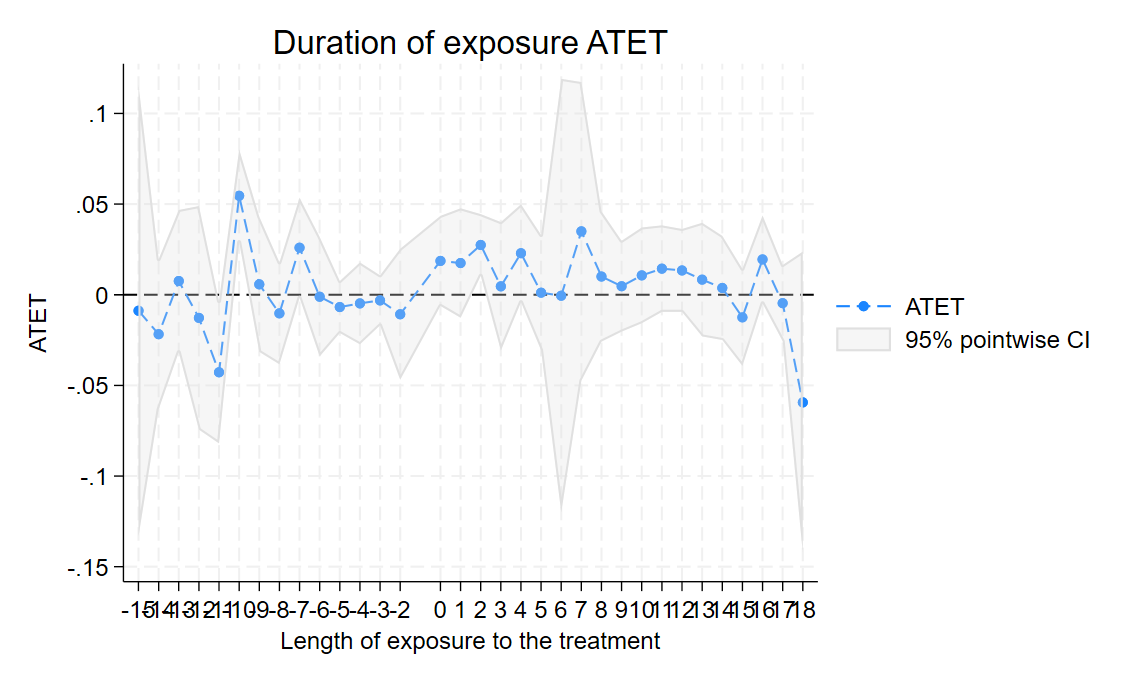


Parallel-trends test (pretreatment time period)

H0: Treatment effects in all the pretreatment periods are zero

chi2(61) = 12250.51

Prob > chi2 = 0.0000

Figure 6. Dynamic graph for 30-day mortality


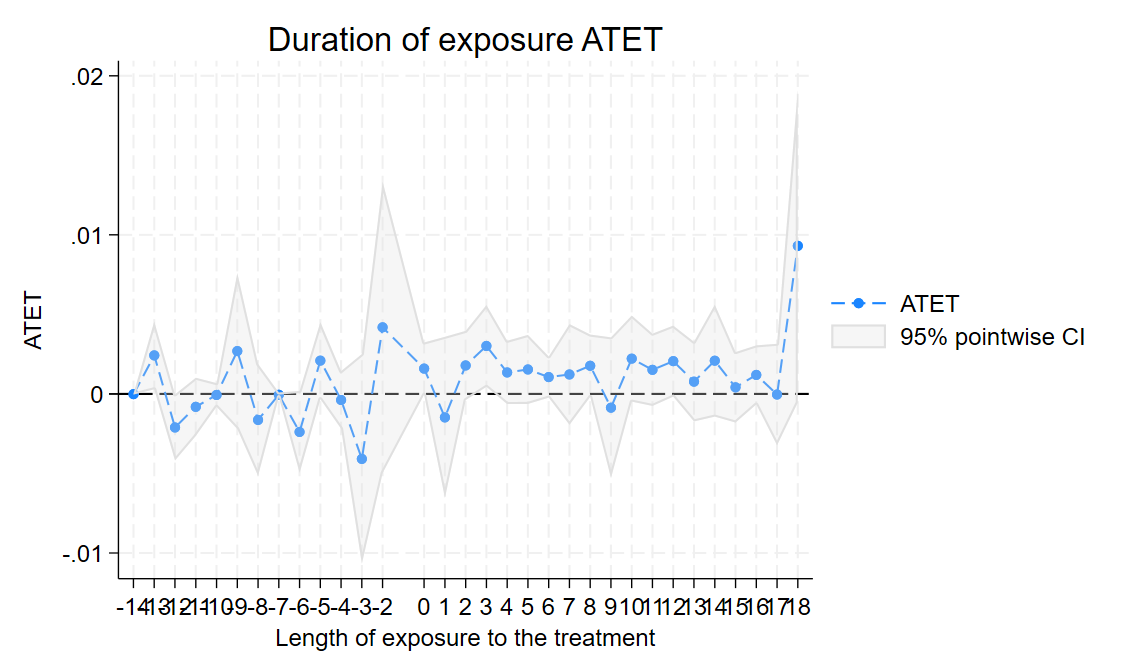


Parallel-trends test (pretreatment time period)

H0: Treatment effects in all the pretreatment periods are zero

chi2(39) = 1288.77

Prob > chi2 = 0.0000

Figure 7. Dynamic graph for 30-day fall-related injury


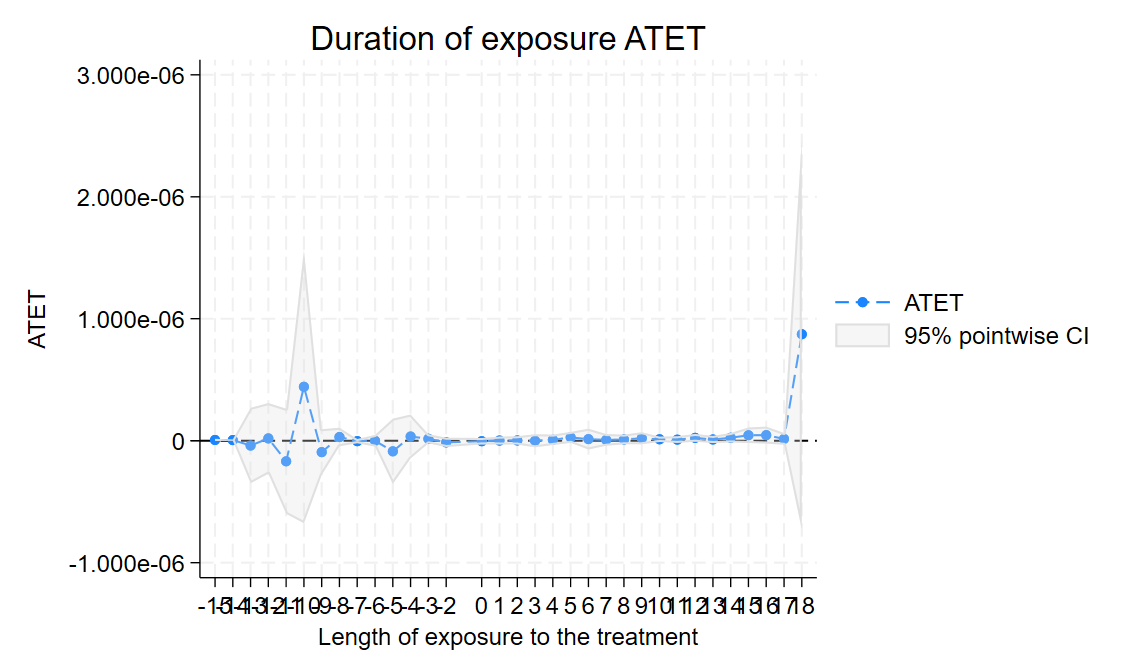


Parallel-trends test (pretreatment time period)

H0: Treatment effects in all the pretreatment periods are zero

chi2(61) = 1058.02

Prob > chi2 = 0.0000

Figure 8. Dynamic graph for ADLs at PAC


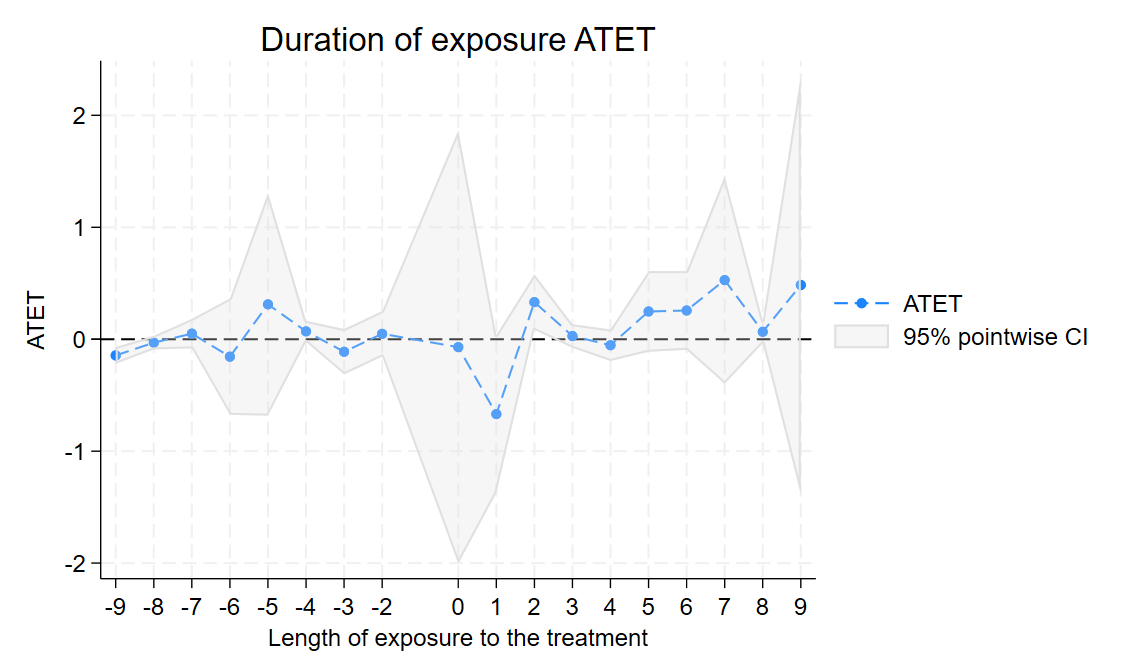


Parallel-trends test (pretreatment time period)

H0: Treatment effects in all the pretreatment periods are zero

chi2(20) = 812.06

Prob > chi2 = 0.0000

Figure 9. Dynamic graph for time to PAC start


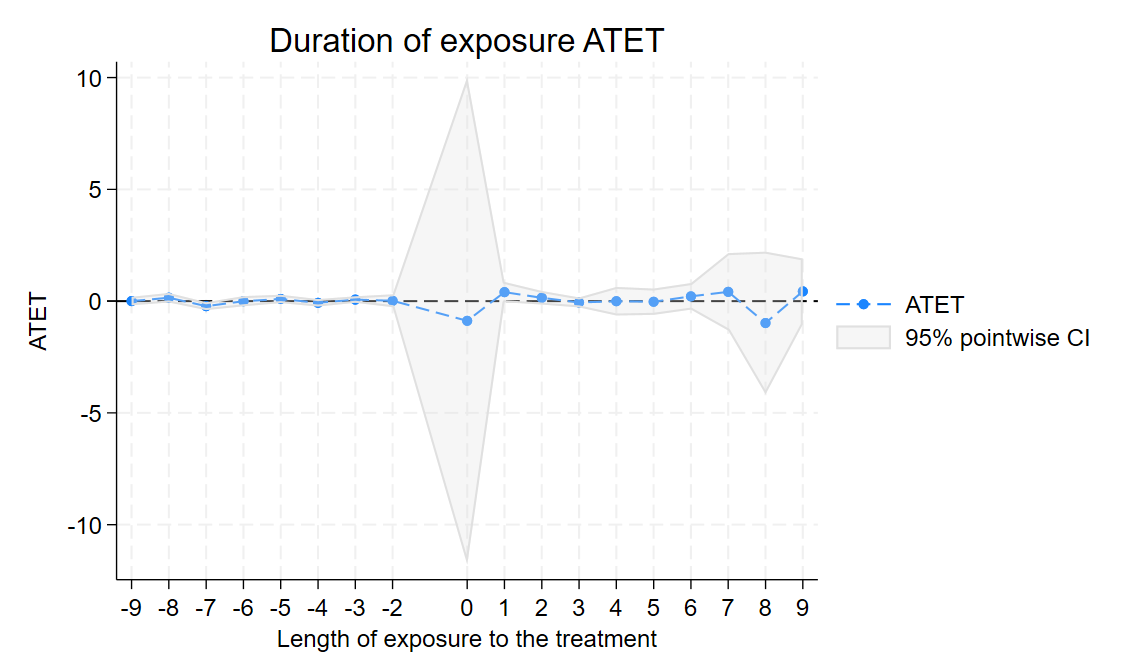


Parallel-trends test (pretreatment time period)

H0: Treatment effects in all the pretreatment periods are zero

chi2(20) = 475.97

Prob > chi2 = 0.0000

Figure 10. Dynamic graph for ADLs to PAC (tertile analysis)


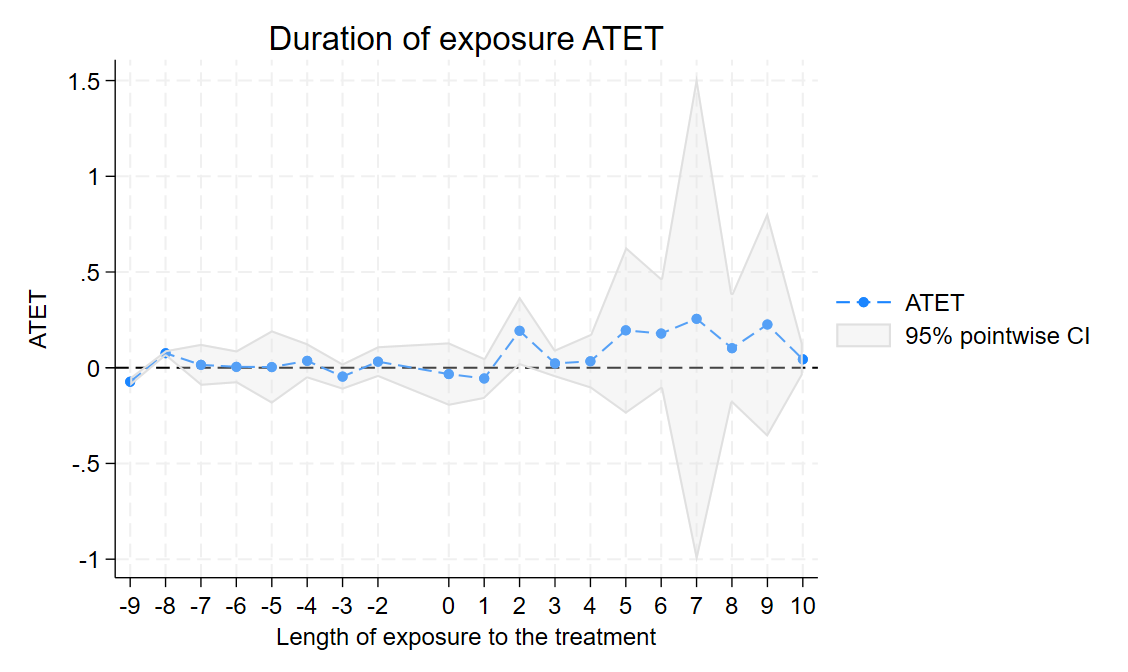


Parallel-trends test (pretreatment time period)

H0: Treatment effects in all the pretreatment periods are zero

chi2(20) = 1864.75

Prob > chi2 = 0.0000

Figure 11. Dynamic graph for time to PAC start (tertile analysis)


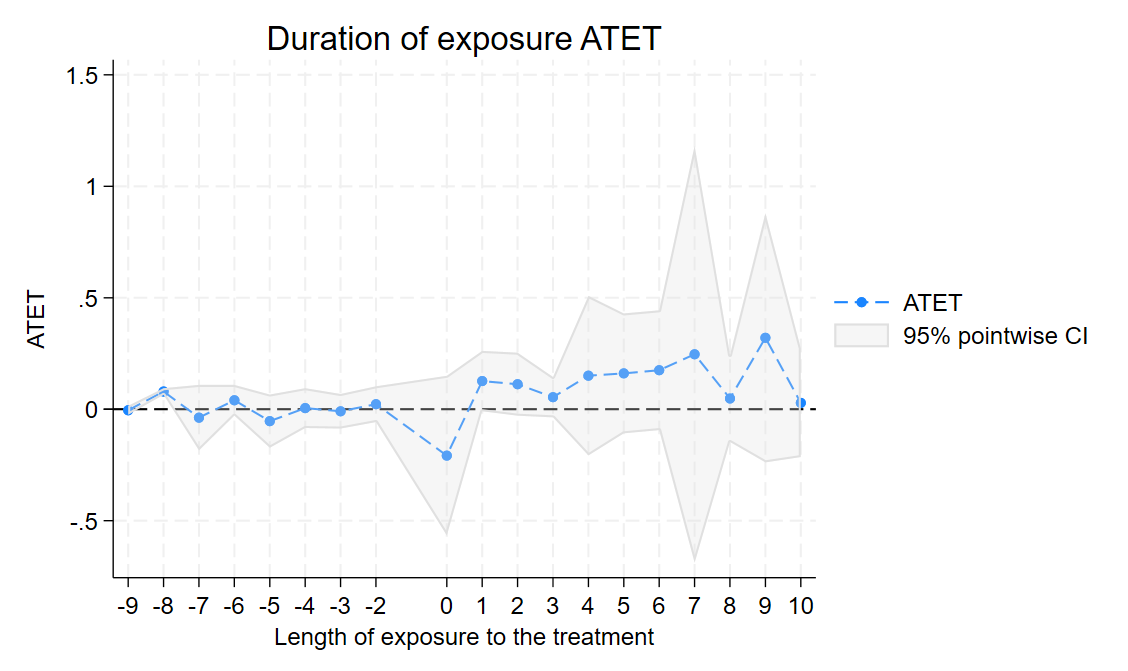


Parallel-trends test (pretreatment time period)

H0: Treatment effects in all the pretreatment periods are zero

chi2(20) = 1521.25

Prob > chi2 = 0.0000

Table A1. **Characteristics of closed hospitals**

|  |  | Mean (SD) / % |  |
| --- | --- | --- | --- |
| Mean no. annual admissions 4 years prior to closure |  | 521.2 (442.6) |  |
| Mean no. annual admissions 1 year prior to closure |  | 242.2 (266.3) |  |
| Mean no. post-acute swing-bed days 1 year prior to closure (N=11) |  | 78.4 (70.4) |  |
| Mean distance to nearest hospital (miles) |  | 17.1 (14.2) |  |
| Mean distance to hospital in county in year prior to closure (N=31) |  | 3.7 (2.3) |  |
| Mean distance to hospital in county in year after closure (N=17) |  | 5.0 (2.6) |  |

Table A2. **Numbers of admissions to nearest hospital after rural hospital closure**

| **Hospital** |  | **1 year prior** |  | **1 year post** |  | **2 years post** |  | **1-year** $\boldsymbol{\Delta}$ |  | **2-year** $\boldsymbol{\Delta}$ |
| --- | --- | --- | --- | --- | --- | --- | --- | --- | --- | --- |
| 1 |  | 54 |  | 58 |  | 65 |  | 4 |  | 11 |
| 2 |  | 87 |  | 90 |  | 68 |  | 3 |  | -19 |
| 3 |  | 239 |  | 231 |  | 247 |  | -8 |  | 8 |
| 4 |  | 557 |  | 619 |  | 872 |  | 62 |  | 315 |
| 5 |  | 372 |  | 344 |  | 484 |  | -28 |  | 112 |
| 6 |  | 393 |  | 444 |  | 467 |  | 51 |  | 74 |
| 7 |  | 312 |  | 432 |  | 471 |  | 120 |  | 159 |
| 8 |  | 1455 |  | 1395 |  | 1767 |  | -60 |  | 312 |
| 9 |  | 51 |  | 32 |  | 53 |  | -19 |  | 2 |
| 10 |  | 71 |  | 105 |  | 98 |  | 34 |  | 27 |
| 11 |  | 275 |  | 593 |  | 749 |  | 318 |  | 474 |
| 12 |  | 482 |  | 592 |  | 635 |  | 110 |  | 153 |
| 13 |  | 0 |  | 11 |  | 0 |  | 11 |  | 0 |
| 14 |  | 171 |  | 186 |  | 162 |  | 15 |  | -9 |
| 15 |  | 784 |  | 699 |  | 649 |  | -85 |  | -135 |
| 16 |  | 535 |  | 554 |  | 506 |  | 19 |  | -29 |
| 17 |  | 409 |  | 363 |  | 350 |  | -46 |  | -59 |
| 18 |  | 106 |  | 140 |  | 197 |  | 34 |  | 91 |
| 19 |  | 918 |  | 820 |  | 814 |  | -98 |  | -104 |
| 20 |  | 28 |  | 30 |  | 23 |  | 2 |  | -5 |
| 21 |  | 316 |  | 0 |  | 0 |  | -316 |  | -316 |
| 22 |  | 1298 |  | 1304 |  | 1275 |  | 6 |  | -23 |
| 23 |  | 756 |  | 749 |  | 646 |  | -7 |  | -110 |
| 24 |  | 117 |  | 257 |  | 274 |  | 140 |  | 157 |
| 25 |  | 225 |  | 239 |  | 175 |  | 14 |  | -50 |
| 26 |  | 79 |  | 151 |  | 191 |  | 72 |  | 112 |
| 27 |  | 34 |  | 35 |  | 20 |  | 1 |  | -14 |
| 28 |  | 591 |  | 489 |  | 422 |  | -102 |  | -169 |
| 29 |  | 891 |  | 1220 |  | 1465 |  | 329 |  | 574 |
| 30 |  | 634 |  | 0 |  | 0 |  | -634 |  | -634 |
| 31 |  | 31 |  | 33 |  | 0 |  | 2 |  | -31 |
| 32 |  | 146 |  | 137 |  | 149 |  | -9 |  | 3 |
| **Total** |  | 12417 |  | 12352 |  | 13294 |  | -65 |  | 877 |
| **Percent Change (%)** | | | | |  |  |  | 0.5 |  | 7.1 |

*Note*: 1 year prior represents the 12-month period prior to the date of the hospital closure. 1 and 2 years post respectively represent the 12-month and 24-month periods directly following the hospital closure. 1 and 2-year $\Delta$ represent the respective change in the numbers of hospital admissions from 1 year prior to each of 1 year and 2 years post closure.

Table A3. **Numbers of admissions to other hospitals in same county after rural hospital closure**

| **Hospital** |  | **1 year prior** |  | **1 year post** |  | **2 years post** |  | **1-year** $\boldsymbol{\Delta}$ |  | **2-year** $\boldsymbol{\Delta}$ |
| --- | --- | --- | --- | --- | --- | --- | --- | --- | --- | --- |
| 1 |  | 916 |  | 997 |  | 1297 |  | 81 |  | 381 |
| 2 |  | 541 |  | 678 |  | 563 |  | 137 |  | 22 |
| 3 |  | 0 |  | 0 |  | 0 |  | 0 |  | 0 |
| 4 |  | 1147 |  | 1265 |  | 1676 |  | 118 |  | 529 |
| 5 |  | 372 |  | 344 |  | 484 |  | -28 |  | 112 |
| 6 |  | 0 |  | 0 |  | 0 |  | 0 |  | 0 |
| 7 |  | 3446 |  | 4261 |  | 4119 |  | 815 |  | 673 |
| 8 |  | 2230 |  | 2120 |  | 2649 |  | -110 |  | 419 |
| 9 |  | 1059 |  | 1034 |  | 1540 |  | -25 |  | 481 |
| 10 |  | 71 |  | 105 |  | 98 |  | 34 |  | 27 |
| 11 |  | 275 |  | 593 |  | 749 |  | 318 |  | 474 |
| 12 |  | 482 |  | 592 |  | 635 |  | 110 |  | 153 |
| 13 |  | 0 |  | 11 |  | 0 |  | 11 |  | 0 |
| 14 |  | 200 |  | 187 |  | 211 |  | -13 |  | 11 |
| 15 |  | 0 |  | 0 |  | 0 |  | 0 |  | 0 |
| 16 |  | 0 |  | 0 |  | 0 |  | 0 |  | 0 |
| 17 |  | 0 |  | 0 |  | 0 |  | 0 |  | 0 |
| 18 |  | 0 |  | 0 |  | 0 |  | 0 |  | 0 |
| 19 |  | 0 |  | 0 |  | 0 |  | 0 |  | 0 |
| 20 |  | 0 |  | 0 |  | 0 |  | 0 |  | 0 |
| 21 |  | 0 |  | 0 |  | 0 |  | 0 |  | 0 |
| 22 |  | 0 |  | 0 |  | 0 |  | 0 |  | 0 |
| 23 |  | 0 |  | 0 |  | 0 |  | 0 |  | 0 |
| 24 |  | 729 |  | 790 |  | 920 |  | 61 |  | 191 |
| 25 |  | 0 |  | 0 |  | 0 |  | 0 |  | 0 |
| 26 |  | 79 |  | 151 |  | 191 |  | 72 |  | 112 |
| 27 |  | 0 |  | 0 |  | 0 |  | 0 |  | 0 |
| 28 |  | 0 |  | 0 |  | 0 |  | 0 |  | 0 |
| 29 |  | 891 |  | 1220 |  | 1465 |  | 329 |  | 574 |
| 30 |  | 0 |  | 0 |  | 0 |  | 0 |  | 0 |
| 31 |  | 31 |  | 34 |  | 29 |  | 3 |  | -2 |
| 32 |  | 183 |  | 157 |  | 186 |  | -26 |  | 3 |
| **Total** |  | 12652 |  | 14539 |  | 16812 |  | 1887 |  | 4160 |
| **Percent Change (%)** | | |  |  |  |  |  | 14.9 |  | 32.9 |

*Note*: 1 year prior represents the 12-month period prior to the date of the hospital closure. 1 and 2 years post respectively represent the 12-month and 24-month periods directly following the hospital closure. 1 and 2-year $\Delta$ represent the respective change in the numbers of hospital admissions from 1 year prior to each of 1 year and 2 years post closure.

Figure A1. **Differential changes in comorbidities after rural hospital closures**


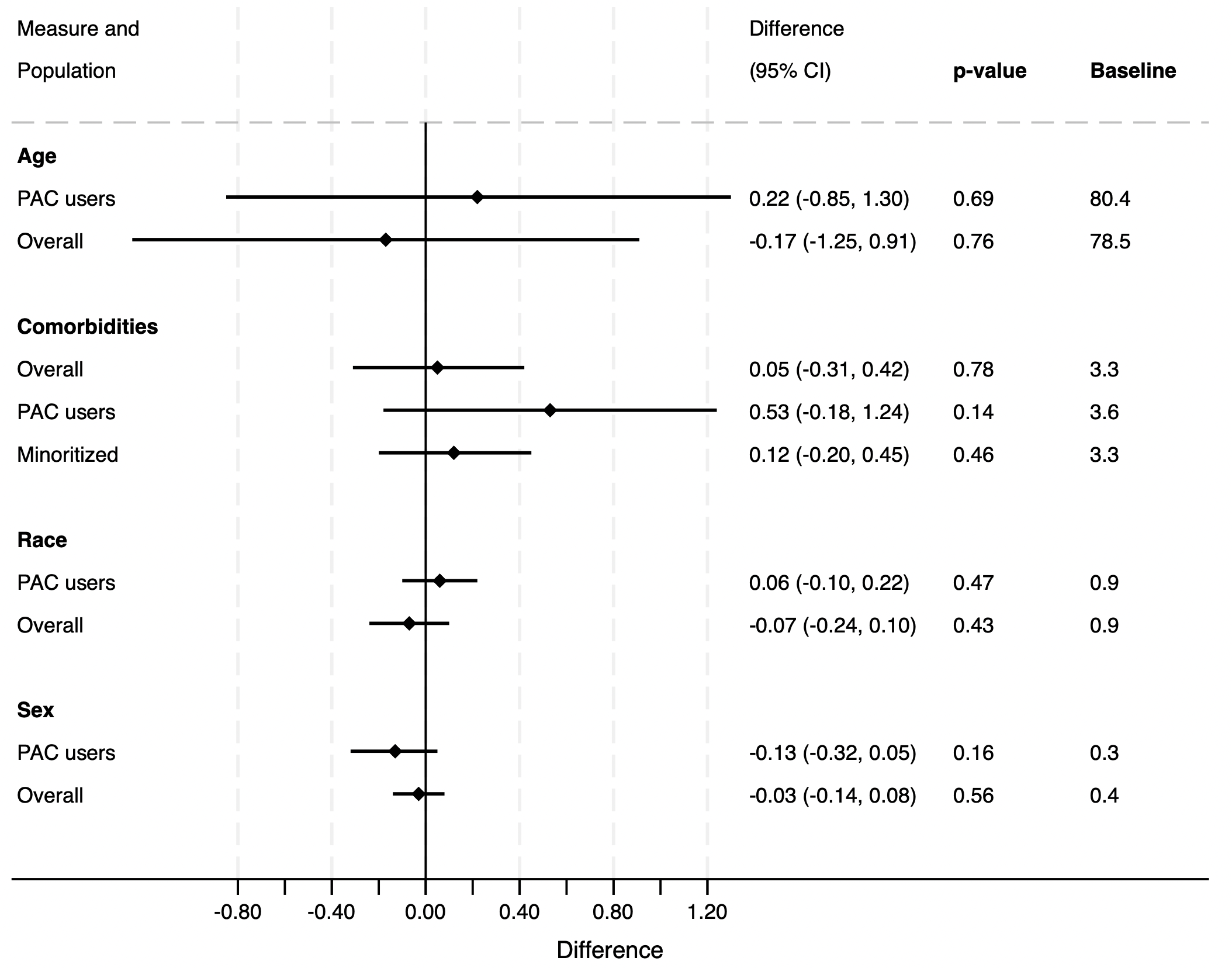


Figure A2. **Differential changes in untrimmed distances to hospitals after rural hospital closures**


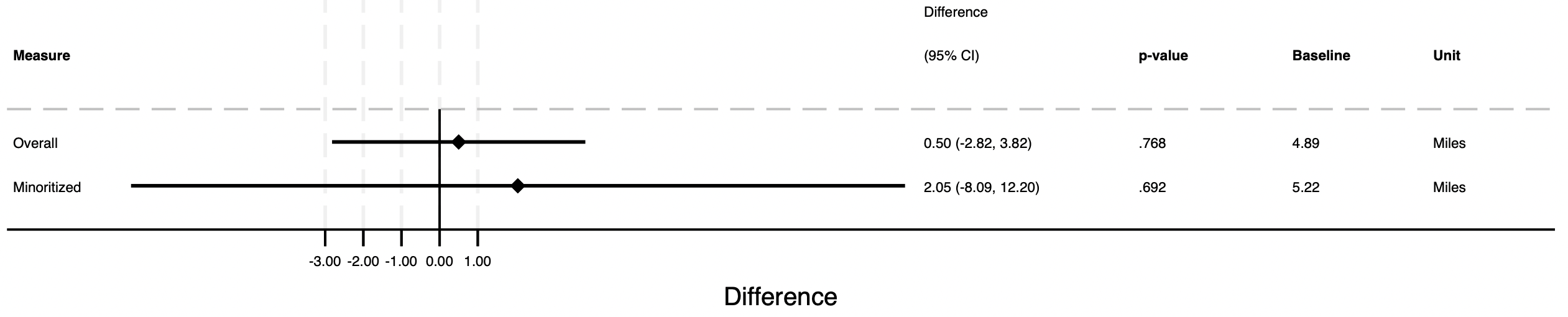


Table A4. **Sensitivity results for associations with rural hospital closures**

|  |  | Coef. |  | 95% CI |  | *p* |  | Baseline |
| --- | --- | --- | --- | --- | --- | --- | --- | --- |
| Hospital that closed was <50 miles of nearest hospital |  |  |  |  |  |  |  |  |
| Distance to hospital |  | 0.02 |  | (-0.69, 0.73) |  | 0.96 |  | 4.50 |
| Length of stay |  | -0.19 |  | (-0.50, 0.13) |  | 0.25 |  | 4.32 |
| 30-day readmission |  | -0.05 |  | (-0.11, 0.02) |  | 0.14 |  | 18.3 |
| 30-day FRI |  | 0.00 |  | (0.00, 0.00) |  | 0.53 |  | 1.35 |
| 30-day mortality |  | 0.00 |  | (0.00, 0.00) |  | 0.84 |  | 0.32 |
| Hospital that closed was not <50 miles of nearest hospital |  |  |  |  |  |  |  |  |
| Distance to hospital |  | 1.21 |  | (-0.48, 2.90) |  | 0.16 |  | 4.24 |
| Length of stay |  | 0.23 |  | (-0.50, 0.95) |  | 0.54 |  | 3.98 |
| 30-day readmission |  | 0.10 |  | (0.00, 0.19) |  | 0.045 |  | 18.0 |
| 30-day FRI |  | 0.00 |  | (0.00, 0.00) |  | 0.15 |  | 1.31 |
| 30-day mortality |  | 0.00 |  | (0.00, 0.00) |  | 0.15 |  | 0.29 |
| Hospitals with swing beds (all individuals) |  |  |  |  |  |  |  |  |
| ADLs at PAC |  | -0.45 |  | -0.64, -0.25 |  | <0.001 |  | -0.15 |
| Time to PAC start |  | 0.01 |  | -0.01, 0.04 |  | 0.36 |  | 0.02 |
| Hospitals without swing beds (all individuals) |  |  |  |  |  |  |  |  |
| ADLs at PAC |  | 0.00 |  | -0.08, 0.09 |  | 0.94 |  | -0.05 |
| Time to PAC start |  | -0.05 |  | -0.13, 0.04 |  | 0.31 |  | 0.08 |
| Hospitals with large Medicare populations |  |  |  |  |  |  |  |  |
| Distance to hospital |  | 0.95 |  | (-1.47, 3.37) |  | 0.44 |  | 4.26 |
| Length of stay |  | 0.23 |  | (-0.48, 0.94) |  | 0.52 |  | 4.00 |
| 30-day readmission |  | 0.01 |  | (-0.08, 0.11) |  | 0.81 |  | 18.0 |
| 30-day FRI |  | 0.00 |  | (-0.02, 0.01) |  | 0.54 |  | 1.31 |
| 30-day mortality |  | 0.00 |  | (-0.05, 0.05) |  | 0.99 |  | 0.29 |

*Note*: “Coef.” indicates coefficient, which represent the change in the outcome after closure. For instance, the coefficient for the “Distance to hospital” model for “Hospital that closed was within 50 miles of nearest hospital” represents a 0.02 increase in the distance to hospital (p=0.96) after rural hospital closures. The coefficient for the 30-day readmission outcomes represents the percentage-point change in the likelihood of a readmission after rural hospital closures.

1. Falvey JR, Murphy TE, Gill TM, Stevens-Lapsley JE, Ferrante LE. Home Health Rehabilitation Utilization Among Medicare Beneficiaries Following Critical Illness. *J Am Geriatr Soc.* 2020;68(7):1512-1519.

2. Stata. Heterogeneous difference in differences (DID). Stata. <https://www.stata.com/new-in-stata/heterogeneous-difference-in-differences/>. Published 2024. Accessed May 28, 2024.
